# Preparation of psoralen polymer–lipid hybrid nanoparticles and their reversal of multidrug resistance in MCF-7/ADR cells

**DOI:** 10.1080/10717544.2018.1464084

**Published:** 2018-04-25

**Authors:** Qingqing Huang, Tiange Cai, Qianwen Li, Yinghong Huang, Qian Liu, Bingyue Wang, Xi Xia, Qi Wang, John C. C. Whitney, Susan P. C. Cole, Yu Cai

**Affiliations:** a College of Pharmacy, Jinan University, Guangzhou, China;; b College of Life Sciences, Liaoning University, Shenyang, China;; c Guangzhou Guoyu Pharmaceutical Technology Co., Ltd, Guangzhou, China;; d GuangzhouJiayuan Pharmaceutical Technology Co., Ltd, Guangzhou, China;; e Department of Biochemistry & Biomedical Sciences, McMaster University, Hamilton, Canada;; f Division of Cancer Biology & Genetics, Queen’s University Cancer Research Institute, Kingston, Canada;; g Cancer Research Institute of Jinan University, Guangzhou, China

**Keywords:** Breast cancer, PSO-PLN, optimal formulation, MCF-7/ADR cells, multidrug resistance

## Abstract

Multidrug resistance (MDR) is the leading cause of failure for breast cancer in the clinic. Thus far, polymer–lipid hybrid nanoparticles (PLN) loaded chemotherapeutic agents has been used to overcome MDR in breast cancer. In this study, we prepared psoralen polymer–lipid hybrid nanoparticles (PSO-PLN) to reverse drug resistant MCF-7/ADR cells *in vitro and in vivo*. PSO-PLN was prepared by the emulsification evaporation-low temperature solidification method. The formulation, water solubility and bioavailability, particle size, zeta potential and entrapment efficiency, and *in vitro* release experiments were optimized in order to improve the activity of PSO to reverse MDR. Optimal formulation: soybean phospholipids 50 mg, poly(lactic-co-glycolic) acid (PLGA) 15 mg, PSO 3 mg, and Tween-80 1%. The PSO-PLN possessed a round appearance, uniform size, exhibited no adhesion. The average particle size was 93.59 ± 2.87 nm, the dispersion co-efficient was 0.249 ± 0.06, the zeta potential was 25.47 ± 2.84 mV. *In vitro* analyses revealed that PSO resistance index was 3.2, and PSO-PLN resistance index was 5.6, indicating that PSO-PLN versus MCF-7/ADR reversal effect was significant. Moreover, PSO-PLN is somewhat targeted to the liver, and has an antitumor effect in the xenograft model of drug-resistant MCF-7/ADR cells. In conclusion, PSO-PLN not only reverses MDR but also improves therapeutic efficiency by enhancing sustained release of PSO.

## 1. Introduction

Multidrug resistance (MDR) is one of the main causes of cancer chemotherapy failure in the clinic (Wu et al., 2014; Alfarouk et al., 2015; Kathawala et al., 2015). In recent years, some studies have shown that MDR may be reversed by combination therapies (Zhao et al., 2015; Meng et al., 2016; Bar-Zeev et al., 2018). Treatment and prevention of cancer with Chinese medicines have a very long history in China (Gottesman et al., 2002; Dai & Fu, 2005; Choi & Yu, 2014; Kibria et al., 2014). In addition, some studies have reported that matrine, quercetin, curcumin, psoralen (PSO), and other traditional Chinese medicine components can cause a significant reversal of MDR (Yuan et al., 2014), but most of them are poorly water-soluble compounds, and have low bioavailability *in vivo* (Wan et al., 2012; Liu et al., 2016), which reduces their MDR reversal activity. As a powder, PSO has poor water solubility, and low bioavailability (van Vlerken et al., 2007). In order to address the above shortcomings, scholars have prepared PSO-PLN and other nano-formulations to improve the bioavailability of PSO.

Recently, the PLN is based on liposome and polymer nanoparticles developed as a new type of drug carrier (Wu, 2016). The PLN is composed of a phospholipid shell and polymer core, thereby providing a core–shell structure with the advantages of both the liposome and polymer nanoparticles (Hao et al., 2008; Yang et al., 2012). The PLN solid polymer core serves as a structural framework and provides mechanical stability, and shape control. It also influences biodegradability, allows for more uniform particle-size distribution, and provides a large surface area (Beija et al., 2012). In addition, the lipid shell of the PLN contributes to its high biological compatibility because its properties are similar to that those of the cell membrane, and thus it can be readily combined with a variety of bioactive molecules (Peetla et al., 2009). The PLN also exhibits enhanced drug loading and encapsulation rate (Souto & Müller, 2008; Zhang et al., 2016). The lipid bilayer structure of PLNs is conducive to adsorbing the drug distributed along the bilayer surface and embeds the hydrophobic region of hydrophilic molecules (Mishra et al., 2015; Pokharkar et al., 2015). PLNs also load and transport both hydrophilic and hydrophobic drugs (van Kuilenburg & Maring, 2013). These nanoparticles can adjust the drug release, provide good stability (Kohandel et al., 2011), and enable the intracellular targeting of delivered drugs (Wang et al., 2010).

The present study was designed to determine the optimal preparation of PSO-PLN by the emulsification evaporation-low temperature solidification method (Liu et al., 2017). Central composite design and response surface optimization methodology (CCD-RSM) was used to determine the optimal formulation, while evaluation indices included encapsulation efficiency and PSO particle size. The properties and the MDR reversal activity of PSO-PLN on drug sensitive MCF-7 and drug resistant MCF-7/ADR cells were also evaluated. In addition, PSO-PLN distribution in Balb/c nude mice was measured at different time points after intravenous injection, and degree of inhibition of drug-resistant tumor growth was measured. We found that drug stability, bioavailability, and physiological compatibility of PSO were all improved when PSO is wrapped or adsorbed on PLN nanostructure.

## 2. Materials and methods

### 2.1 Materials

PSO (HPLC98%, Nanjing Spring and Autumn Biological Engineering Co., Ltd, Jiangsu, China), PLGA (50:50, Jinan Daigang Biomaterial Co., Ltd, Shandong, China), soybean lecithin (injection grade, Shanghai, China), Tween-80 (Aladdin, Shanghai, China), ethanol, and acetone were all of analytical purity, whereas all other reagents were of chromatographic grade. The MCF-7 and MCF-7/ADR cell lines were purchased from Keygen Biotech (Nanjing, Jiangsu, China) and cultured at 37 °C in a humidified incubator with 5% CO_2_ and maintained in RPMI1640 medium (Gibco) supplemented with 10% fetal bovine serum and 1% 100 U/mL penicillin/streptomycin. The cell doubling times were typically 24 h. Balb/c nude mice (16–20 g, female) were obtained from Beijing Huafu Kang Biological Technology Co., Ltd (Beijing,China). All animals were raised in SPF level environment. All animal experiments were conducted with the formal approval of Jinan University Animal Center.

### 2.2 Methods

#### 2.2.1 Preparation of PSO-PLN

PSO-PLNs were prepared using the emulsification evaporation-low temperature solidification method. Both PLGA (15 mg) and PSO (3 mg) were dissolved in acetone to form an organic phase. Soy lecithin (50 mg) and 1% Tween-80 were dissolved in 4% ethanol to form the water phase. After heating the water phase to 75 °C, the organic phase was slowly injected into the aqueous phase and stirred for approximately 1 h at 75 °C. The concentrated emulsion was then added to 15 mL of ice water and stirred at 20 rpm for 1 h. The resulting PSO-PLNs were centrifuged at 1000 rpm for 5 min. The supernatant was filtered through a 0.45 µm membrane filter and stored at 4 °C.

#### 2.2.2 Encapsulation efficiency (EE) of PSO-PLN

EE of PSO-PLN was determined using an ultrafiltration method. PSO-PLN suspension (0.5 mL) was placed in a 10 mL volumetric flask, and added methanol. The suspension was sonicated for 30 min and filtered through a 0.45 µm membrane filter. Total dose (*W*_t_) was determined using high-performance liquid chromatography (HPLC). PSO-PLN suspension (0.5 mL) was centrifuged at 12,000 rpm for 20 min using a super filter (30 kDa, Millipore, Darmstadt, Germany). The supernatant was removed and filtered through a 0.45 µm membrane filter. Then the free drug volume (*W*_f_) was determined using HPLC. Drug EE was calculated using the following formula:

$$EE=(W_{t}-W_{f})/W_{t}\times 100\%$$

#### 2.2.3 Optimization of PSO-PLN

A series of experiments in which a single parameter (temperature, stirring time, PLGA, lipid content, PSO dosage, and Tween-80 concentration) was carried out. These experiments showed that the lipid content, PSO dosage, and PLGA content used in preparation of PSO-PLN were the three most critical factors that influenced the particle size (Y1) and EE (Y2). These were then used as evaluation indexes to select the lipid content (soybean phosphor lipid), PLGA content, and PSO dosage (PSO content) as determined according to the Box–Behnken principle.

#### 2.2.4 Morphology of PSO-PLN

The morphology of PSO-PLN was determined using transmission electron microscopy (TEM). PSO-PLN suspension was dropped using a copper wire with a carbon film to 2% phosphorous acid for 1 min and observed by TEM.

#### 2.2.5 Particle size and zeta potential analysis

Zeta potential, poly dispersity index (PDI), and particle size were determined using a laser particle size analyzer.

#### 2.2.6 *In vitro* release of PSO-PLN

The release of PSO and PSO-PLN was measured using dynamic dialysis. PSO-PLN suspension and PSO solution was added to a dialysis bag with molecular weight cutoff of 8000–14,000.The dialysis bag was then placed in a dissolution apparatus and stirred at 100 rpm at 37 °C. After initiating dialysis, the cumulative release of PSO-PLN and PSO in the media was calculated. Samples were collected at 0.5, 1, 2, 4, 8, 12, 24, 36, and 48 h after the start of dialysis and the PSO content was measured by HPLC.

#### 2.2.7 Fourier transform infrared (FTIR) spectral studies

The chemical integrity of the drug and the polymetric matrix was investigated using FTIR spectra (Perkin-Elmer, Model Spectruml, CA). Samples were crushed with KBr to get the pellets by applying a pressure of 300 kg/cm^2^. FTIR spectra of PSO, blank-PLN, PSO + PLN, and PSO-PLN were scanned in the range between 4000 and 500 cm^−1^.

#### 2.2.8 Resistance index of MCF-7/ADR cells

MCF-7 cells were cultured in logarithmic phase, whereas MCF-7/ADR cells were cultured in a complete medium to prepare the cell suspension. The cell suspension was diluted as needed, and MCF-7 cells were counted and adjusted to 0.5 × 10^4^ cells per well. MCF-7/ADR cells were seeded in 96-well culture plates at 1 × 10^4^ cells per well, and 100 µL of cell suspension was added to each well and the plates placed in an incubator. MCF-7/ADR cells were incubated for 24 h and cultured at 100 µL per well at different concentrations (0.0625, 0.125, 0.25, 0.5, 1, 2, 4, and 8 µg/mL). DOX (0.15, 6.25, 12.5, 25, 50, 100, and 200 µg/mL) was added to the 100 µL/well cells, drug-free control (cells without drug), and no cell control (culture medium only). The cells were incubated for 5 min at 1000 rpm. The supernatant was discarded, and 150 µL of dimethyl sulfoxide was added to the solution. The solution was shaken for 5 min and absorbance (OD) values were read using a microplate reader at a wavelength of 570 nm. Growth inhibition of MCF-7 cells and MCF-7/ADR cells at different DOX concentrations and relative drug resistance of the MCF-7/ADR cells were calculated as follows:

$$\%\;Inhibition=1-({OD}_{treatment}-{OD}_{blank\;group})/({OD}_{control\;group}-{OD}_{blank\;group}\;)\times 100\%.$$

Resistance index = IC_50_ DOX of MCF-7/ADR cells/IC_50_ DOX of MCF-7 cells.

#### 2.2.9 Reversal index of MCF-7/ADR cells

MCF-7/ADR cells were exposed to PSO and PSO-PLN to compare the ability of the two agents to reverse resistance to DOX. DOX (1.57, 3.13, 6.25, 12.5, 25, 50, 100, and 200 µg/mL) was combined with either the PSO solution or the PSO-PLN dispersion solution, and IC50 was detected after 48 h.

Reversal index = IC_50_ (DOX)/IC_50_ (DOX with reversing agent).

#### 2.2.10 *In vivo* fluorescence imaging

To image PSO in live animals, xenografts were first established as follows: MCF-7/ADR cells (1 × 10^7^) were injected subcutaneously on the right side of the armpit of female Balb/c nude mice (4–5 weeks old, 16–20 g in weight) and observed over 2 weeks. The behavior, diet, and defecation patterns of Balb/c nude mice were recorded. When a MCF-7/ADR tumor had grown to 100 mm^3^, the mouse was anesthetized by injection with PSO-PLN and PSO-SLN, respectively, then injection pentobarbital and *in vivo* fluorescence images of the anesthetized Balb/c nude mice were obtained at different time points (0, 1, 2, 4, 8, and 12 h). For *in vivo* fluorescence imaging, near-infrared labeled PSO-SLN and PSO-PLN were obtained by adding indocyanine green (ICG) during the preparation of PSO-SLN and PSO-PLN.

#### 2.2.11 Antitumor experiment

Balb/c nude mice were divided into four groups and treated with phosphate-buffered saline (PBS), DOX (3 mg/kg), DOX + PSO (3 mg/kg), and DOX + PSO-PLN (3 mg/kg), respectively. Animals were administered intravenous injections once every 3 days for 21 days. Weight and diameter of the nude mice were measured, and tumor volume was calculated using Formula (1). Growth of the tumor was calculated using Formula (2).

Tumor inhibition was calculated as follows:

Tumor inhibition = (mean tumor volume of saline group − mean tumor volume of treatment group)/mean tumor volume of saline group.

Tumor volume and tumor growth inhibition were calculated as follows:

(1) $$Tumor\;volume={1/2ab}^{2}$$

(2) $$Tumor\;inhibition\;(\%)=(V_{PBS}-V_{treatment}\;)/V_{PBS}\times 100\%$$

where *a* is tumor diameter, *b* is tumor short diameter, *V*_PBS_ represents the volume of xenograft-bearing mice receiving PBS, and *V*_treatment_ denotes the volume of xenograft-bearing mice receiving DOX, PLN, PSO-PLN nanoparticles.

Nude mice xenografts and various normal tissues were randomly selected from each group, fixed in formalin, and sent to the First Affiliated Hospital of Guangzhou University of Traditional Chinese Medicine Pathological Laboratory for hematoxylin and eosin staining of paraffin sections, and pathological changes recorded.

#### 2.2.12 Data analysis

All measurements were made in three replicates within a single experiment, and the values were expressed as standard deviation (SD). Data were subjected to Student’s *t*-test analysis, and results considered statistically significant when *p* < 0.05.

## 3. Results

### 3.1 Optimization of PSO-PLN

The Box–Behnken response surface method (Sah & Suresh, 2017) is commonly used to explore formulation in pharmaceutical preparations. In this study, we found that the deviation between the measured and predicted values was less than 10%. Therefore, the model established by the Box–Behnken response surface fit was valid. Response-Expert 8.0.6 software (Minneapolis, Minnesota, USA) was used for regression model analysis. Linear regression and binary polynomial regression were performed using soybean phospholipids, PLGA, and PSO, as independent variables and particle size and EE as dependent variables. Goodness (*r*) and confidence (*P*) served as model judgment criteria. Quadratic multiple regression models were as follows:

$$Y_{\text{1}}=\text{64}.0\text{6}-\text{5}.\text{2}0A+0.\text{26}B+\text{1}0.\text{36}C-\text{8}.\text{7}0AB-0.\text{82}AC-\text{7}.\text{22}BC+\text{4}.\text{84}A^{\text{2}}+\text{1}.0\text{2}B^{\text{2}}-\text{6}.\text{48}C^{\text{2}}\left(R^{\text{2}}=0.\text{9737}\right)$$

*A*, *B*, *C*, *A*^2^, and *C*^2^ (*p* < .01) are significant, and the remaining ones are non-significant.

$$Y_{\text{2}}=\text{7}0.\text{92}-\text{1}.\text{29}A+\text{1}0.\text{96}B+\text{8}.\text{96}C+\text{9}.\text{94}AB+0.0\text{13}AC-\text{3}.\text{97}BC+0.\text{75}A^{\text{2}}-\text{5}.\text{99}B^{\text{2}}-\text{3}.\text{84}C^{\text{2}}\left(R^{\text{2}}=0.\text{91}00\right)$$

*B*1, *AC*, *B*^2^, *C*^2^ (*p* < .01) are significant, and the remaining ones are non-significant.

**Figure 1.**
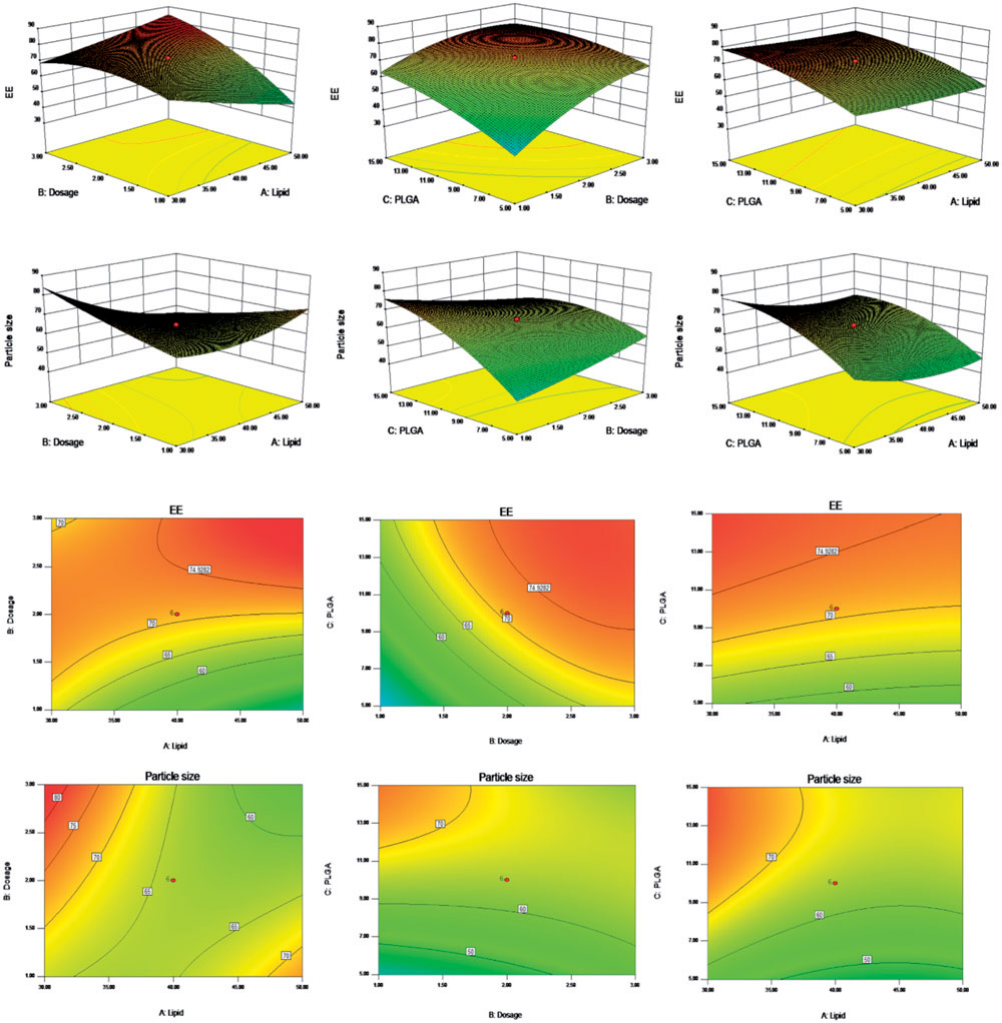
Response surfaces of the lipid content, PSO dosage, and PLGA content of PSO-PLN and its response values. The preparation was made thrice, and the results were compared with prescribed ones. The deviation between the experimentally obtained values and predicted ones was less than 10%, indicating that the model fit was good.

Response surface analysis was conducted using the Design-Expert 8.0.6 software (Minneapolis, Minnesota, USA) to map the 3D effect of package size, EE, and other factors through the results of binary polynomial model fitting. Figure 1 shows the various relationships between the factors and their influence on particle size and EE.

### 3.2 Characterization of PSO-PLN

**Figure 2.**
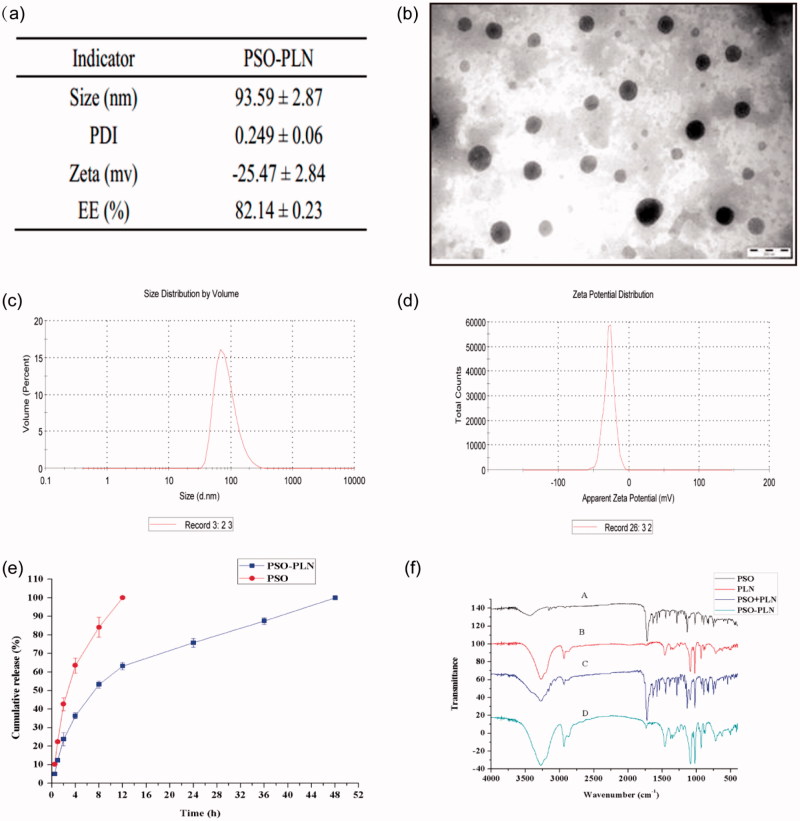
(a) Particle properties of PSO-PLN. (b) Transmission electron microscopy image of PSO-PLN after optimal formulation. (c) Properties of the optimal particle size of 93.59 ± 2.87 nm. (d) Properties of the optimal particle size of Zeta potential of −25.47 ± 2.84 mV. (e) *In vitro* drug (PSO) release profiles from PSO solution and PSO-PLN. Release was measured at neutral (pH 7.4) conditions at 37 ± 0.5 °C. Each point represents the mean (±SD) based on triplicate measurements. (f) FTIR spectra of (A) PSO, (B) PLN, (C) PSO + PLN, (D) PSO-PLN.

As seen from Figure 2(b), TEM experiments showed that PSO-PLNs exhibited uniform size, spherical shape, and absence of adhesion. Figure 2(c) shows that zeta potential of PSO-PLN was closely related to the stability of PSO-PLN at −25.47 ± 2.84 mV. When electrostatic repulsion between particle sizes reached more than 20 mV, PSO-PLN possessed a particle size of 93.59 ± 2.87 nm in Figure 2(d), and only a single peak appeared in the spectrum. The PDI of the sample was 0.249 ± 0.06 (<0.3), indicating the decentralized particle size of the nanoparticles.

### 3.3 *In vitro* release of PSO-PLN

Figure 2(e) shows that PSO solution was released completely within the first 4 h, whereas the *in vitro* release of PSO-PLN displayed a significant sustained-release effect, with approximately 90% cumulative release in 36 h. In the first 4 h, PSO-PLN underwent a burst-release process, resulting in a cumulative release of approximately 50%. It is possible that the drug was adsorbed onto the surface of PSO-PLN in the dialysis medium and retained in the PSO-PLN structure, resulting in a slow release effect.

### 3.4 Fourier transform infrared (FTIR) spectral studies

FTIR analysis was used to characterize any chemical (formation of chemical bonds) changes that occurred in the polymer due to the addition of drug during the synthesis reaction. Figure 2(f) shows the FTIR spectra of PSO, blank-PLN, PSO + PLN, and PSO-PLN. The presence of PSO in PLN can be analyzed through its characteristic carbonyl absorption peak. It can be seen from Figure 2(f) that carbonyl can be observed in the PSO monomer and physical mixture. But there is no obvious carbonyl absorption peaks in the infrared scanning pattern of PSO-PLN, blank-PLN, which indicated that PSO may form a new crystal in PLN.

### 3.5 Cytotoxicity study on drug resistance

**Figure 3.**
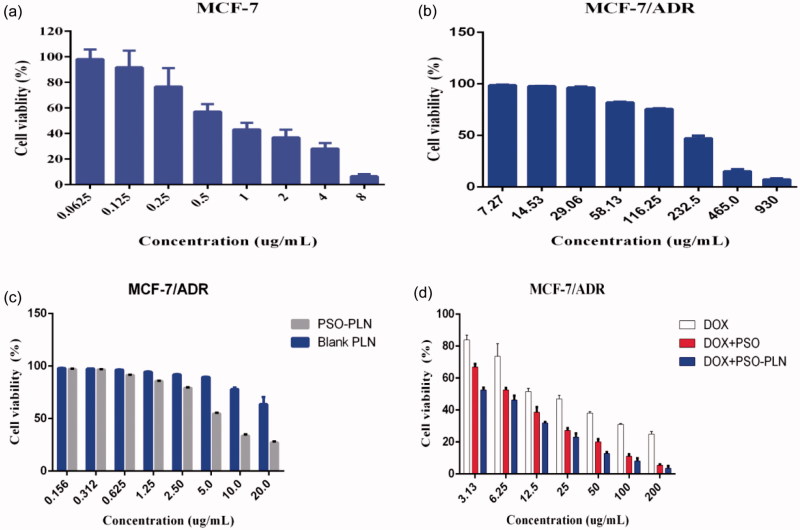
The cytotoxicity of various formulations against MCF-7 cells and MCF-7/ADR cells for 48 h. (a) Cell viability for MCF-7 cells treated with DOX at different concentrations ranged from 0.0615 to 8 μg/mL. (b) Cell viability for MCF-7/ADR cells treated with PSO at different concentrations ranged from7.27 to 930 μg/mL. (c) Cell viability for MCF-7/ADR cells treated with PSO-PLN and blank-PLN with different concentrations ranged from 0.156 to 20 μg/mL. (d) *In vitro* cytotoxicity of free DOX, free DOX + PSO, and DOX + PSO-PLN against MCF-7/ADR cells. The DOX was at a concentration ranged from 3.13 to 200 μg/mL. The cytotoxity assay was performed by MTT assay. Results are expressed as mean ± SD (*n* = 6).

The IC_50_ values for DOX for the MCF-7 and MCF-7/ADR cell lines were 0.9166 and 24.03 µg/mL, respectively (Figure 3(a, d)), yielding a relative drug resistance for the MCF-7/ADR cell line of 26.22. In order to investigate the reversal index, the anti-proliferative effect of various formulations in MCF-7/ADR cells was determined using the classical MTT method. At first, we evaluated the potential cytotoxity of PSO, blank-PLN, and PSO-PLN. Based on our results, PSO, blank-PLN, and PSO-PLN had no obvious toxicity to MCF-7/ADR cells in the range of 7.27–29.06 μg/mL, 0.156–2.5 μg/mL, and 0.156–0.625 μg/mL (Figure 3(b, c)). Thus, the reversed concentration of PSO and PSO-PLN was finally determined to be 0.625 μg/mL. According to the data presented in Figure 3(d), the IC50 values of free DOX, DOX + PSO, and DOX + PSO-PLN were 24.03 μg/mL, 7.296 μg/mL, and 4.275 μg/mL, respectively. The reversal index (RRI) of PSO and PSO-PLN were 3.2 and 5.6, respectively. These data indicate that co-delivery of the anticancer drug and nanoparticles might improve the reversal activity.

### 3.6 Fluorescence imaging of xenograft-bearing nude mice

**Figure 4.**
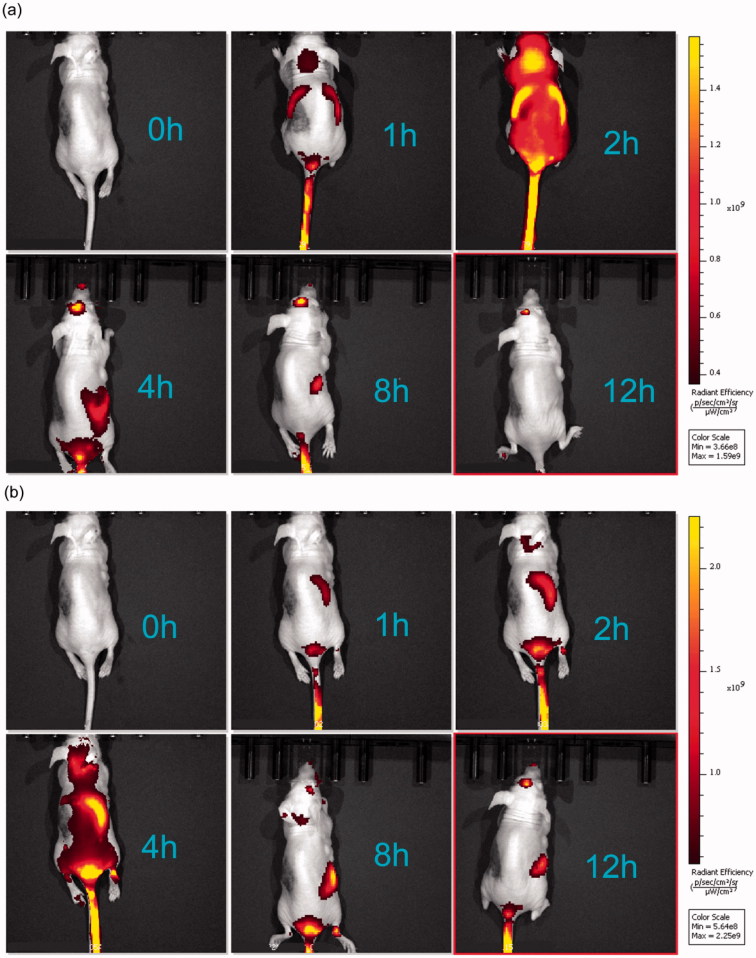
*In vivo* targeted fluorescence imaging of xenograft mice bearing MCF-7/ADR cells treated with DOX + PSO-SLN (a) or DOX + PSO-PLN (b) at 0, 1, 2, 4, 8,12 h. PSO-SLN and PSO-PLN were obtained by adding ICG during the preparation of PSO-SLN and PSO-PLN.

Figure 4 shows live fluorescence images of the mice treated with PSO-SLN, PSO-PLN. All mice were treated with DOX. In comparison with the DOX + PSO-SLN group, PSO required 1 h to reach the liver, 2 h for rapid liver metabolism, and 4 h to reach the kidneys 8 h after gradual metabolism. In comparison with the DOX + PSO-PLN group, the drug in the DOX + PSO-PLN group needed 1 h to reach the liver and 4–8 h for liver accumulation before reaching the kidneys for metabolism and drug accumulation. When compared with the DOX + PSO-SLN group, residency time of the drug in the liver and kidneys was significantly prolonged in the PSO-PLN group. This finding indicated that PSO-PLN caused a sustained-release effect and extended the length of time PSO remains in the body.

### 3.7 Effects of DOX and/or DOX + PSO or DOX + PSO-PLN on nude mice bearing MCF-7/ADR xenograft tumors

**Figure 5.**
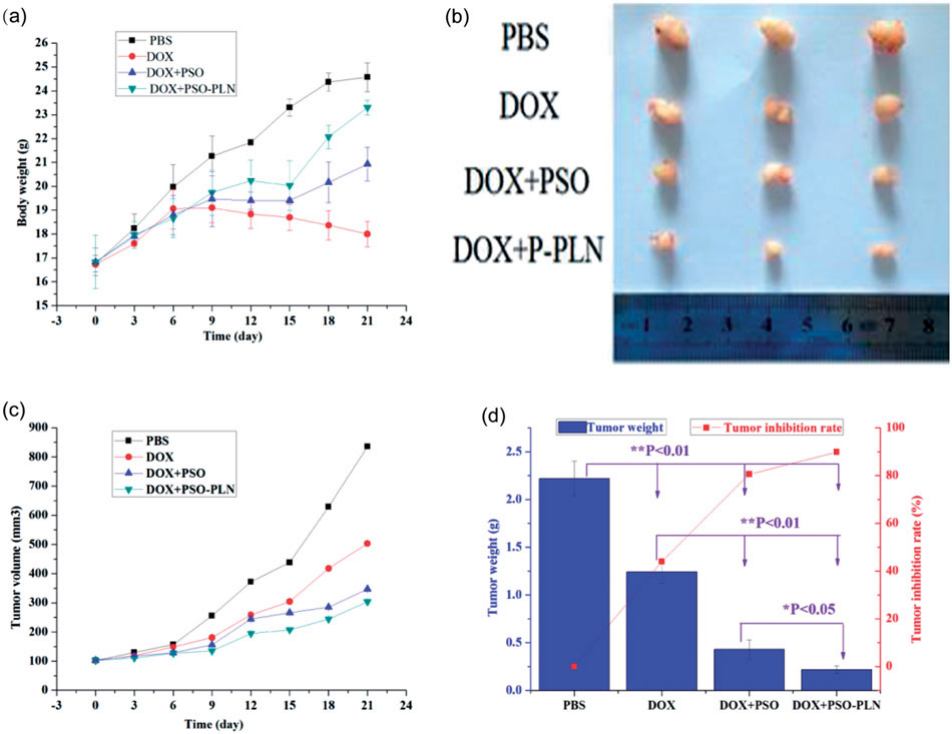
Evaluation of antitumor efficacy *in vivo*. (a) Body weight change of mice during various treatments. (b) Photographs of representative tumors from mice bearing MCF-7/ADR xenografts after treatment. (c) Changes in tumor volume of MCF-7/ADR tumor xenograft bearing nude mice from day 1 to day 21. (d) Tumor inhibition rate after antitumor treatment. Data points represent means ± SD, *p < 0.05 is considered statistically significant, *n* = 4 per group.

Before the first administration of DOX and/or DOX + PSO or DOX + PSO-PLN the dimension of the MCF-ADR xenograft tumors were measured using vernier calipers, and tumor volumes were calculated by Formula. DOX and/or DOX + PSO or DOX + PSO-PLN were administered once every 3 days for 21 days (7 times in total). Tumor volume was then calculated. As shown in Figure 5(a), the body weight of the DOX + PSO-PLN treated group increased to some extent relative to that of the PBS, DOX + PSO, and DOX + PSO-PLN treated groups. The average weight of the DOX-treated group decreased, indicating that DOX caused toxicity. Mice in the DOX-treated group were also thinner after exposure to DOX. Tumors were removed at the end of experiments and three mice selected from each group for imaging (Figure 5(b)). Figure 5(c) shows that at the end of experiments, MCF-ADR tumors from the PBS treated control group were the largest among all the groups. Tumor growth was inhibited to a certain extent in the DOX and DOX + PSO groups. The tumor volumes from the DOX + PSO-PLN group were the smallest, indicating that PSO-PLN enhances the tumor inhibitory effect of DOX to a certain extent. As shown in Figure 5(d), inhibition of tumor growth of the DOX, DOX + PSO, and DOX + PSO-PLN groups reached 44.0%, 80.6%, and 89.9%, respectively.

**Figure 6.**
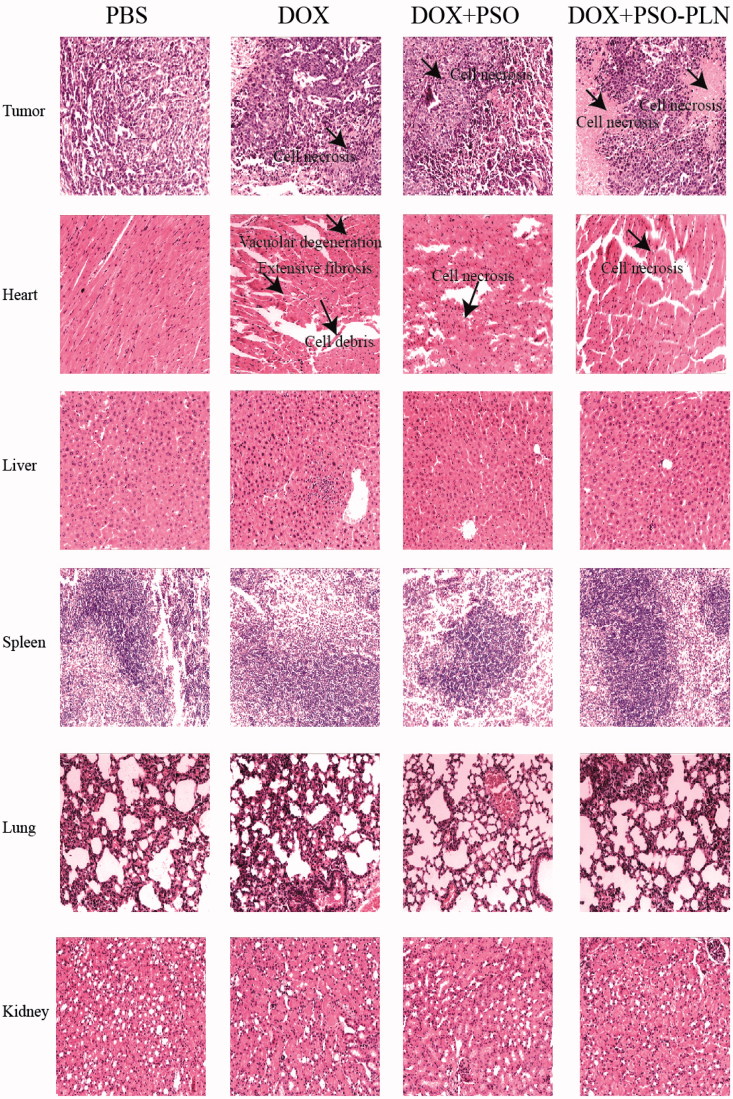
Pathological sections of tissues from mice bearing MCF-7/ADR xenografts after treatment. The organs sections were stained with hematoxylin and eosin. Scale bar is 5 μm.

The effects of the different treatments on tissue histology of the tumor-bearing mice are shown in Figure 6 and can be summarized as follows:

Tumors: Among all groups, the DOX + PSO-PLN group exhibited the most significant degree of tumor necrosis.

Spleen: None of the four treatment groups showed any splenicstructural abnormalities. Membrane morphology and trabecular structure were also normal. No red pulp or white pulp congestion were observed.

Lung: None of the four treatment groups showed any structural abnormalities in the lung. Alveoli and elastic fibers exhibited no atrophy, necrosis, excessive expansion, nor emphysema. Alveolar septa were evenly abundant and without any trace of emphysema.

Kidneys: None of the four treatment groups showed any structural abnormalities in the kidneys. Glomerular morphology was normal and without either inflammatory cell in filtration or abnormal cell proliferation. Renal tubular epithelial cells were normal and showed no degeneration or necrosis. The tube or interstitial infiltrations of inflammatory cells was not observed.

Heart: In the DOX-treated group, heart cells appeared flaky and exhibited a disordered arrangement and vacuolar degeneration. In the DOX + PSO, and the DOX + PSO-PLN treated groups, heart cells appeared necrotic. Necrosis was most evident in the DOX-treated group. No lesions were found in the PBS group, which also exhibited neatly arranged myocardial fibers, a rich cytoplasm, and an absence of heart fiber fracture, hypertrophy, atrophy, edema, inflammatory cell infiltration, and thickening of the pericardium.

Liver, the PBS, DOX, DOX + PSO, and DOX + PSO-PLN treated groups all showed a normal liver structure. The liver cells showed no evidence of steatosis, jaundice, necrosis, or inflammatory cell infiltration. The portalarea was uncongested in all four groups.

These results indicate that under the conditions test, only the DOX-treated mice exhibited evidence of myocardial toxicities.

## 4. Conclusions and discussion

Breast cancer is the leading cause of death from cancer in women worldwide (ACS, 2016). Chemotherapy represents one key treatment modality for the clinical management of breast cancer, but MDR frequently occurs in the course of cancer chemotherapy and eventually results in its failure (Holohan et al., 2013). Earlier studies have suggested that PSO reverses MDR in cancer cells (Hsieh et al., 2014; Wang et al., 2016), but PSO features poor water solubility and low bioavailability (Ciaravino et al., 2001). To improve upon these shortcomings, the application of PLN as a drug delivery system has become a topic of considerable interest in recent years. The application of nanotechnology is effective for treating drug-resistant cancers as it increases pharmacological activity and reduced systemic toxicity of chemotherapy drugs (Yuan et al., 2016). Herein, we developed PSO-PLN combination with free DOX to treat drug-resistant breast cancer. PLGA and other packaging materials can be used effectively for hydrophobic drugs (Wu, 2016). PSO is a hydrophobic drug, whereas PLGA, as a polymer, can effectively contain PSO. In the present study, the optimal PSO-PLN prepared possessed a core–shell structure, PLGA, and a PSO core. It also possessed an improved mechanical stability, small and uniform particle size distribution, large surface area, and is easily biodegradable. Soybean lecithin was located on the outer layer of PLGA and PSO, forming a structure similar to the phospholipid bilayer. As a result, soybean lecithin can bind to a variety of bioactive molecules (Li et al., 2017).

The particle diameter of PSO-PLN was 93.59 ± 2.87 nm with a PDI (0.249 ± 0.06) (Figure 2(a)). Small particle size is expected to increase cellular internalization and improve intracellular concentrations of drugs (Duan et al., 2017). Nanoparticles with diameters (∼100 nm) can accumulate in tumor cells *via* the leaky tumor vasculature and prolong blood circulation in tumor tissues through the enhanced permeability and retention (EPR) effect (Kibria et al., 2014). The zeta potential plays an important role in physical stability and biological applications of nanoparticles (PSO-PLN, 25.47 ± 2.84 mV). Moreover, PSO was released from PLN in good controlled manner (Figure 2(e)). This sustained release pattern was coincided with those of *in vivo* fluorescence imaging results (Figure 4).

To investigate the effects of different formulations on cell viability, we calculated the IC_50_ value (Figure 3). According to the IC_50_ values, the PSO-PLN showed a high cytotoxic effect when combination with DOX. The results suggest that combination of nanoparticles and anticancer drugs increased therapeutic efficacy and selectivity to drug-resistant cancer cells. After *in vitro* analysis, the antitumor efficacy of PSO-PLN was further validated in nude mice bearing MCF-7/ADR cell tumors. As shown in Figure 5(a), PSO or PSO-PLN could reduce doxorubicin-induced weight loss (**p < 0.01), which demonstrating that PSO or PSO-SLN could reduce DOX-induced toxicity to some extent. The efficacy of decreasing DOX-induced toxicity also manifests in myocardial tissue sections (Figure 6). More importantly, DOX + PSO-PLN had a significant effect on controlling tumor progression (∼250 mm^3^) compared with the free DOX group (∼450 mm^3^). The antitumor efficacy of PSO-PLN is attributed to its nanosized and controlled drug release kinetics. Besides, the presence of soybean lecithin on the outer shell improved blood circulation, which may enhance tumor accumulation.

In conclusion, using PSO-PLN extends the effect of PSO by producing a sustained release compared with free PSO. *In vivo* fluorescence imaging showed that PSO-PLN exhibited a liver-targeting effect. This coincided with those of in vitro release investigation of PSO-PLN. Furthermore, PSO-PLN contributed to cytotoxicity of DOX *in vitro* and *e*nhanced reversal activity in drug resistant MCF-7/ADR xenograft model compared with PSO. These results indicated that PSO-PLN could have significant advantages for the treatment of drug-resistant breast cancer and warrants further investigation.
